# Repeatability of anterior segment measurements by optical coherence tomography combined with Placido disk corneal topography in eyes with keratoconus

**DOI:** 10.1038/s41598-020-57926-7

**Published:** 2020-01-24

**Authors:** Domenico Schiano-Lomoriello, Valeria Bono, Irene Abicca, Giacomo Savini

**Affiliations:** IRCCS – Fondazione Bietti, Rome, Italy

**Keywords:** Corneal diseases, Tomography

## Abstract

Corneal tomography is an important tool to identify and follow up eyes with keratoconus. Our study evaluate the repeatability of the automatic measurements provided in keratoconic eyes by a new anterior-segment optical coherence tomographer (AS-OCT) combined with Placido-disk topography (MS-39, CSO) and assess their agreement with the corresponding measurements taken with a rotating Scheimpflug camera combined with Placido-disk topography (Sirius, CSO). Mean simulated keratometry, posterior and total corneal power, total corneal astigmatism, corneal asphericity, thinnest corneal thickness, epithelial thickness, corneal diameter, and aqueous depth were evaluated. Repeatability was assessed using test–retest variability, the coefficient of variation, and the intraclass correlation coefficient; agreement was assessed by the 95% limits of agreement. Good repeatability was achieved for most parameters. Moderate repeatability was found for total corneal astigmatism measurements. The repeatability of mean simulated keratometry and total corneal power measurements worsened with more severe stages of keratoconus with a statistically significant relationship between the individual coefficient of variation and corneal power values. Agreement with the Scheimpflug camera was moderate for aqueous depth and thinnest corneal thickness and poor for most other measured parameters. The good repeatability of automatic measurements suggests the new AS-OCT device to be a viable option in clinical practice of eyes with keratoconus.

## Introduction

Keratoconus is a non-inflammatory ectasic disease characterized by progressive thinning and steepening and an apical cone-shaped protrusion of the cornea^1,2^. The introduction of corneal topography first and corneal tomography later enabled researchers to develop several methods to identify keratoconic eyes^3–7^. Accurate corneal imaging is now highly desirable to diagnose keratoconus, especially in its earlier stages, when visual acuity is still good. Modern anterior segment imaging techniques, such as Scheimpflug photography and optical coherence tomography (OCT), have significantly improved our ability to identify eyes with keratoconus, as they also provide pachymetric data and posterior corneal curvature measurements. Although anterior corneal surface parameters may be sufficient to discriminate between normal eyes and eyes with clinical keratoconus, using the data from both corneal surfaces as well as corneal thickness increases precision, sensitivity, specificity, and accuracy, thus significantly improving the ability to differentiate between normal eyes and subclinical keratoconus suspect eyes^7^.

One of the main limitations of Scheimpflug imaging is the low resolution and relatively poor quality of anterior segment scans. In this regard, anterior segment OCT (AS-OCT) is known to produce better images with higher definition. The higher resolution has made it possible, for example, to measure the corneal epithelial thickness^8–10^. A parameter that has never been evaluated by Scheimpflug cameras.

The purpose of this study is to evaluate the repeatability of measurements provided in keratoconic eyes by a new AS-OCT device combined with Placido-disk corneal topography (MS-39, CSO, Florence, Italy) and to assess their agreement with the corresponding measurements taken with a rotating Scheimpflug camera combined with Placido-disk topography (Sirius, CSO).

## Results

The study enrolled 44 participants, 29 men and 15 women (mean age 45.1 years ± 16.75 standard deviation[SD], range 17 to 79 years), affected by keratoconus. This was classified as belonging to Stage I in 31 eyes (72.1%), Stage II in 11 eyes (25.6%) and stage III in 1 eye (2.3%).

Table 1 shows the test–retest repeatability, coefficient of variation (CoV), and Intra-class correlation coefficient (ICC) for the parameters measured by the AS-OCT device. A CoV lower than 1%, revealing excellent repeatability, was obtained for many parameters: simulated keratometry (Sim-K), total corneal power (TCP), corneal diameter and aqueous depth (AQD). A low CoV (<5%) was also obtained with other parameters, such as posterior keratometry (posterior K), thinnest corneal thickness and central epithelial thickness. Repeatability was lower for the total corneal astigmatism (TCA) magnitude, whose CoV was 16.58%. An ICC >0.9 was calculated for all parameters, thus revealing good repeatability for each.

**Table 1:** Repeatability analysis of the measurements provided by AS-OCT combined with Placido corneal topography.

	Test-retest repeatability	Coefficient of Variation (%)	ICC
Sim-K (D)	0.80	0.62	0.990
Posterior K (D)	0.36	2.20	0.950
Total corneal power (D)	0.66	0.51	0.995
TCA magnitude (D)	1.53	16.58	0.957
Anterior corneal asphericity (Q-value)	0.29	—	0.951
Corneal diameter (mm)	0.19	0.59	0.967
Thinnest corneal thickness (µ)	14.18	1.06	0.974
Central epithelial thickness (µ)	4.34	3.17	0.921
Aqueous depth (mm)	0.03	0.34	0.999

The repeatability of SimK and TCP measurements worsened with more severe stages of keratoconus. Linear regression disclosed a statistically significant relationship between the individual COV (i.e., the CoV obtained from 3 measurements of the same eye) and SimK (r = 0.500, p = 0.0007) as well as TCP (r = 0.3441, p = 0.0257). A statistically significant relationship was detected also for the anterior corneal asphericity in the 8.0 mm zone (Q-value of the anterior surface), whose individual SD was correlated with the stage of keratoconus severity, as determined by SimK (r = 0.5494, p = 0.0001). Such a relationship was not observed for the other parameters.

Table 2 shows the mean values for each parameter measured by the two devices. Compared to the Scheimpflug camera, the AS-OCT Placido topographer provided higher Sim-K and TCP values. For these parameters, the mean difference was, respectively, 0.63 ± 0.72 D (p < 0.0001) and 1.59 ± 1.44 D (p < 0.0001). Accordingly, agreement was poor, especially for TCP. Statistically and clinically significant differences were found for posterior corneal power (less negative with the AS-OCT Placido topographer), anterior Q-value (less negative with the AS-OCT Placido topographer) and corneal diameter (shorter with the AS-OCT Placido topographer). For all these values, agreement was poor.

**Table 2:** Mean values from the 2 devices in keratoconus patients.

Parameter	AS-OCT– Placido Topographer	Scheimpflug Camera–Placido Topographer		P value
	Mean ± SD	Mean ± SD	95% LoA	
Simulated K (D)	46.74 ± 2.82	46.11 ± 2.63	−0.78, + 2.03	<0.0001
Posterior K (D)	−5.95 ± 0.56	−6.71 ± 0.57	−0.35, + 1.87	<0.0001
Total corneal power (D)	47.05 ± 3.23	45.46 ± 2.43	−1.24, + 4.41	<0.0001
TCA power (D)	3.39 ± 2.81	3.26 ± 2.61	−1.63, + 1.88	0.6078
TCA (D @ axis)	0.40 @ 85	0.66 @ 91	NA	NA
Anterior Q-value	−0.41 ± 0.45	−0.67 ± 0.46	−0.42, + 0.93	<0.0001
Thinnest corneal thickness (mm)	478.91 ± 41.43	472.33 ± 42.09	−22.32, + 35.48	0.0055
Central epithelial thickness (mm)	48.88 ± 5.49	—	—	—
Corneal diameter (mm)	11.86 ± 0.36	12.53 ± 0.98	−2.35, + 1.02	<0.0001
Aqueous depth (mm)	3.24 ± 0.35	3.18 ± 0.33	−0.04, + 0.16	<0.0001

Statistically significant differences were also found for aqueous depth and thinnest corneal thickness, both slightly higher with the AS-OCT Placido topographer, although the mean difference (0.06 ± 0.05 mm and 6.58 ± 14.74 µ, respectively) in these cases has little impact from a clinical point of view. Agreement for these two parameters was moderate. Finally, TCA magnitude was slightly higher with the AS-OCT Placido topographer, but in the case of this parameter the difference was not statistically significant. Table 2 also shows the mean value of TCA calculated by vector analysis, which includes both magnitude and axis; with both devices a very low astigmatism was obtained due to vector summation, which makes this information of little value.

## Discussion

The present study primarily shows two new findings: (1) the measurements provided by the AS-OCT Placido topographer show good repeatability in eyes with keratoconus and (2) the agreement of these measurements with those provided by a Scheimpflug camera combined with Placido disc corneal topography is low for most parameters.

The good repeatability of the values measured by the AS-OCT Placido topographer is of utmost importance, as it enables an accurate follow-up to detect keratoconus progression and the effects of procedures like cross-linking. All parameters revealed high repeatability, as the ICC was >0.9. The worse values were observed for TCA magnitude, whose CoV was 16.58%, but this is not surprising, as the repeatability of corneal astigmatism magnitude measurements is relatively low with all instruments, even in healthy eyes. Our data are similar to those previously reported by other authors^11–15^.

We had already investigated the repeatability of measurements provided by the same AS-OCT Placido topographer in eyes with no pathologies and in eyes that had undergone excimer laser surgery^16^. Compared to these eyes, the repeatability in eyes with keratoconus was lower for most parameters.

For example, the CoV of SimK increased from 0.16% (healthy unoperated eyes) to 0.62% (keratoconus); similarly the CoV of posterior K increased from 0.39% to 2.20% and that of the thinnest corneal thickness from 0.32% to 1.06%. In contrast, the repeatability was slightly better for aqueous depth. A lower repeatability of anterior segment measurements in eyes with keratoconus has been previously reported for different technologies^11,17^. The irregularity of the corneal shape and curvature in keratoconus is the most likely explanation for the reduced repeatability (accordingly, the repeatability of aqueous depth measurements is not worse).

Repeatability was good also for central epithelial thickness, a parameter that has been found to play a role in the diagnosis of early stages of keratoconus^18^.

Agreement between measurements generated by the OCT-based topographer and the Scheimpflug-based topographer was low and this warrants some comments. The corneal power, as calculated by both SimK and TCP, was higher with OCT than with the Scheimpflug technique. Since SimK may be entered into intraocular lens (IOL) power formulas, different optimized formula constants would be required for the two instruments to obtain a zero mean prediction error. On the other hand, TCP is not routinely used for IOL power calculation; however it is interesting to observe that the mean difference with this value was even higher (1.59 D) than in the case of SimK. Such a difference reflects the difference in the posterior corneal curvature, as the OCT device measured a flatter (less negative) back surface than the Scheimpflug camera. We do not have an explanation for this finding, which was not observed in healthy and post-refractive surgery eyes^16^. However, future studies on keratoconus corneal power should take this difference into account.

Differences with respect to aqueous depth and thinnest corneal thickness were less significant from a clinical point of view, whereas a large discrepancy was observed for the corneal diameter, which was significantly larger with the Scheimpflug camera combined with the Placido topographer. This difference was not observed in our previous study^16^, where the “adjusted corneal diameter” setting was selected.

In the present study we did not select this setting and, as a consequence, the Scheimpflug system overestimated the corneal diameter. This difference should be considered when using corneal diameter to predict phakic lens sizing.

This study has some limitations. First, our sample included only a minority of cases with severe keratoconus, where we may expect worse results. Second, we did not investigate all the parameters provided by the two devices, but only those commonly used.

In conclusion, our data suggest that the new AS-OCT Placido topographer can be used in eyes with keratoconus to measure anterior segment parameters with good repeatability. Due to significant differences and poor agreement for most values, we do not recommend using the OCT-based and Scheimpflug-based devices interchangeably.

## Methods

### Participants

This prospective study enrolled patients with keratoconus. The diagnosis of keratoconus was made by one of the authors (DSL) on the basis of typical slit-lamp (Fleischer rings or Vogt striae) and topographic findings (asymmetric bow-tie pattern with or without skewed axes).

Eyes were classified as belonging to Stage I when the keratometric power of the cornea was <48 diopters (D), Stage II when it was between 48 and 53 D, and Stage III when it was >53 D^19^. We excluded eyes with corneal scarring or previous surgery (including cross-linking), eyes with subclinical keratoconus (i.e., eyes with forme fruste keratoconus and keratoconus suspects, as defined by Klyce)^20^, eyes with a history of any corneal disease, and contact lens use in the month preceding enrolment. If both eyes were affected with keratoconus, only one was randomly selected. The study was performed in accordance with the ethical standards stated in the 1964 Declaration of Helsinki and approved by the IRCCS G.B. Bietti Foundation clinical research ethics committee. All patients provided informed consent.

### Instruments

The MS-39 (software version 3.6) uses spectral domain OCT (SD-OCT) and Placido-disc corneal topography to measure the anterior segment of the eye. Once auto-calibration is performed, the device acquires one Placido top-view image and a series of 25 SD-OCT radial scans at a wavelength of 840 nm, with an axial resolution of 3.5 nm, a transverse resolution of 35 nm and a maximum depth of 7.5 mm. Each scan is 16-mm long and includes 1024 A-scans. The ring edges are detected on the Placido image so that height, slope, and curvature data can be calculated using the arc-step method with conic curves. Profiles of the anterior cornea, posterior cornea, anterior lens, and iris are derived from the SD-OCT scans. Data for the anterior surface from the Placido image and SD-OCT scans are merged using a proprietary method. All other measurements for internal structures (posterior cornea, anterior lens, and iris) are derived solely from SD-OCT data. The repeatability of measurements performed with this device in healthy eyes has already been reported^16^.

The Sirius (software version 3.2) combines a single rotating Scheimpflug camera and a Placido disk corneal topographer. The scanning process acquires a series of 25 Scheimpflug images (meridians) and 1 Placido top-view image. Technical details have been previously described^17^.

### Procedures

The same procedures as previously described were carried out^16^. In the case of AS-OCT, three repeated consecutive measurements were taken by the same experienced examiner (DSL) to assess repeatability. The patients were asked to sit back after each measurement, and the device was realigned before the subsequent measurement. In the case of Sirius, only one measurement was acquired. The starting sequence of the devices was drawn at random.

### Measured parameters

We evaluated the same measurements recently investigated in normal and post-refractive surgery eyes^16^. They include the mean Sim-K (calculated using the standard keratometric index of 1.3375), posterior corneal curvature, TCP (obtained by ray tracing through the anterior and posterior surfaces and a 3-mm diameter entrance pupil), total corneal astigmatism (TCA, analyzed with and without vector analysis)^21^, anterior and posterior corneal asphericity in the 8.0 mm zone (calculated as the Q-value), corneal diameter, thinnest corneal thickness, epithelial thickness and aqueous depth (AQD, i.e., the axial distance between the corneal endothelium and the anterior surface of the lens). The details of each measured parameter have been previously described^13^. With the exception of epithelial thickness, the corresponding values provided by the Sirius were analyzed to assess agreement.

### Statistical analysis

The term repeatability was based on the definition of the International Organization for Standardization^22^, which considers it a part of accuracy. Accuracy includes trueness and precision. Trueness is defined as the inverse of bias and is achieved by comparing the measurement result with the accepted reference (conventional true) value. Precision is defined as the inverse of statistical uncertainty and is expressed in terms of the standard deviation (SD). Precision has two conditions: repeatability and reproducibility. Under repeatability conditions, all factors (the operator, the equipment used, the equipment calibration, the environment and the elapsed time between measurements) are considered constant and do not contribute to the variability of the measurement result. Under reproducibility conditions, those factors can change. Repeatability and reproducibility are the two extremes of precision.

Repeatability was investigated by means of the following methods:

1. Intra-session test-retest variability (also known as repeatability or limits of repeatability). This was obtained by multiplying the pooled within-subject SD (s_w_) by 2.77^23^. According to this value, it can be expected that the difference between two measurements for the same subject will be less than 2.77 s_w_ for 95% of pairs of observations.
2. Coefficient of variation (CoV). This was calculated as the s_w_ divided by the mean of the measurements and expressed as a percentage. The CoV cannot be calculated for measurements showing both positive and negative values^24^.
3. Intra-class correlation coefficient (ICC). This is the ratio of the between-subjects variance to the sum of the pooled within-subject variance and the between-subjects variance. The ICC, which gets close to 1.0 when there is no variance between repeated measurements, was automatically calculated using SPSS software (version 22) with the 2-way mixed model and absolute agreement. ICCs ranging from 0 to 1 are usually classified as follows: ICC less than 0.75 = poor agreement; ICC between 0.75 and 0.90 = moderate agreement and ICC 0.90 and greater = high agreement^25^.

The level of agreement between the 3 instruments was evaluated according to the method described by Bland and Altman^26^, who suggested plotting the differences between measurements (y axis) against their mean (x axis). Bland and Altman plots allow us to investigate the existence of any systematic difference between measurements (i.e., fixed bias). The mean difference is the estimated bias, and the standard deviation (SD) of the differences measures the random fluctuations around this mean. The 95% limits of agreement (LoAs) were defined as means ±1.96 SD of the differences between the two measurement techniques. In addition, a paired t-test was used to compare the mean values measured by the two devices (with MS-39, only the first scan was used for this purpose).

The sample size was calculated in order to achieve a minimum 15% confidence in the estimate. As shown by McAlinden *et al*.^27^, at least 43 eyes had to be enrolled in each group to satisfy this condition.

## Data Availability

The data used to support the findings of this study are available from the corresponding author upon request.
